# Celastrol alleviates renal fibrosis by upregulating cannabinoid receptor 2 expression

**DOI:** 10.1038/s41419-018-0666-y

**Published:** 2018-05-22

**Authors:** Ming Tang, Xu Cao, Kun Zhang, You Li, Quan-you Zheng, Gui-qing Li, Qian-hui He, Shu-jing Li, Gui-lian Xu, Ke-qin Zhang

**Affiliations:** 1 Department of Nephrology, Southwest Hospital, Army Medical University (Third Military Medical University), Chongqing, 400038 China; 2 Department of Immunology, Army Medical University (Third Military Medical University), Chongqing, 400038 China

## Abstract

Renal fibrosis is the final manifestation of various chronic kidney diseases, and no effective therapy is available to prevent or reverse it. Celastrol, a triterpene that derived from traditional Chinese medicine, is a known potent anti-fibrotic agent. However, the underlying mechanisms of action of celastrol on renal fibrosis remain unknown. In this study, we found that celastrol treatment remarkably attenuated unilateral ureteral obstruction (UUO)-induced mouse renal fibrosis. This was evidenced by the significant reduction in tubular injury; collagen deposition; accumulation of fibronectin, collagen I, and α-smooth muscle actin; and the expression levels of pro-fibrotic factors *Vim*, *Cola1*, and *TGF-β1* mRNA, as well as inflammatory responses. Celastrol showed similar effects in a folic acid-induced mouse renal fibrosis model. Furthermore, celastrol potentiated the expression of the anti-fibrotic factor cannabinoid receptor 2 (CB2R) in established mouse fibrotic kidney tissues and transforming growth factor β1 (TGF-β1)-stimulated human kidney 2 (HK-2) cells. In addition, the CB2R antagonist (SR144528) abolished celastrol-mediated beneficial effects on renal fibrosis. Moreover, UUO- or TGF-β1-induced activation of the pro-fibrotic factor SMAD family member 3 (Smad3) was markedly inhibited by celastrol. Inhibition of Smad3 activation by an inhibitor (SIS3) markedly reduced TGF-β1-induced downregulation of CB2R expression. In conclusion, our study provides the first direct evidence that celastrol significantly alleviated renal fibrosis, by contributing to the upregulation of CB2R expression through inhibiting Smad3 signaling pathway activation. Therefore, celastrol could be a potential drug for treating patients with renal fibrosis.

## Introduction

Chronic kidney disease (CKD) is currently recognized as an important public health problem worldwide, and its treatment has remained a daunting task^1^. Renal interstitial fibrosis, which is regarded as the final common pathway in all forms of CKDs, is characterized by inflammatory cell infiltration, activated myofibroblast accumulation, deposition of extracellular matrix (ECM), and tubular atrophy^2,3^. These pathologies eventually lead to end-stage renal disease^4^ and, unfortunately, therapeutic strategies for inhibiting or reversing renal fibrosis remain limited. Therefore, there is an urgent need to develop satisfactory therapeutic drugs for renal fibrosis.

Celastrol (C_29_H_38_O_4_) is a bioactive compound extracted from the traditional Chinese medicinal plant *Tripterygium wilfordii* Hook F (TwHF, thunder God Vine)^5^. Increasing evidence suggests that celastrol possesses potent anti-inflammatory, antioxidant, and immunosuppressive properties^6,7^. It has beneficial effects on tumors and autoimmune and inflammatory diseases^5,8^. In addition, celastrol has been shown to suppress cardiac and pulmonary fibrosis, and ameliorate disruption of the endothelial barrier^9–11^. However, the effect of celastrol on renal fibrosis has not been studied.

Transforming growth factor β1 (TGF-β1) is known as a vital pro-fibrotic factor in renal fibrosis^12^. As the downstream mediator of TGF-β1, SMAD family member 3 (Smad3) plays a critical role in renal fibrosis by targeting fibrogenic genes and tissue inhibitor of metalloproteinase 1^13^. In contrast, the activation of cannabinoid receptor 2 (CB2R), a specific metabotropic receptor of the endocannabinoid system (ECS), reduces inflammation in cerebral ischemic injury, acute myocardial infarction, nephropathy, and cystitis^14–17^. Moreover, CB2R activation also attenuates hepatic and skin fibrosis in mouse models^18,19^. Activating of CB2R with agonist inhibits the development of renal fibrosis, and a CB2R antagonist blunts this beneficial anti-fibrotic effect in a unilateral ureteral obstruction (UUO) mouse model^20^. This indicates that CB2R may also serve as a potential target for the treatment of renal fibrosis. However, the relationship between Smad3 signaling and CB2R expression during renal fibrosis development is unclear.

In the present study, we explored the potential therapeutic effects of celastrol on renal fibrosis and the correlation of the mechanisms of action of celastrol and the activation of anti-fibrotic factor CB2R using UUO-induced and folic acid (FA)-induced mouse renal fibrosis models. We found that celastrol inhibited the progression of renal fibrosis by upregulating CB2R expression through inhibiting Smad3 signaling pathway activation.

## Results

### Celastrol treatment attenuates renal fibrosis

**Fig. 1:**
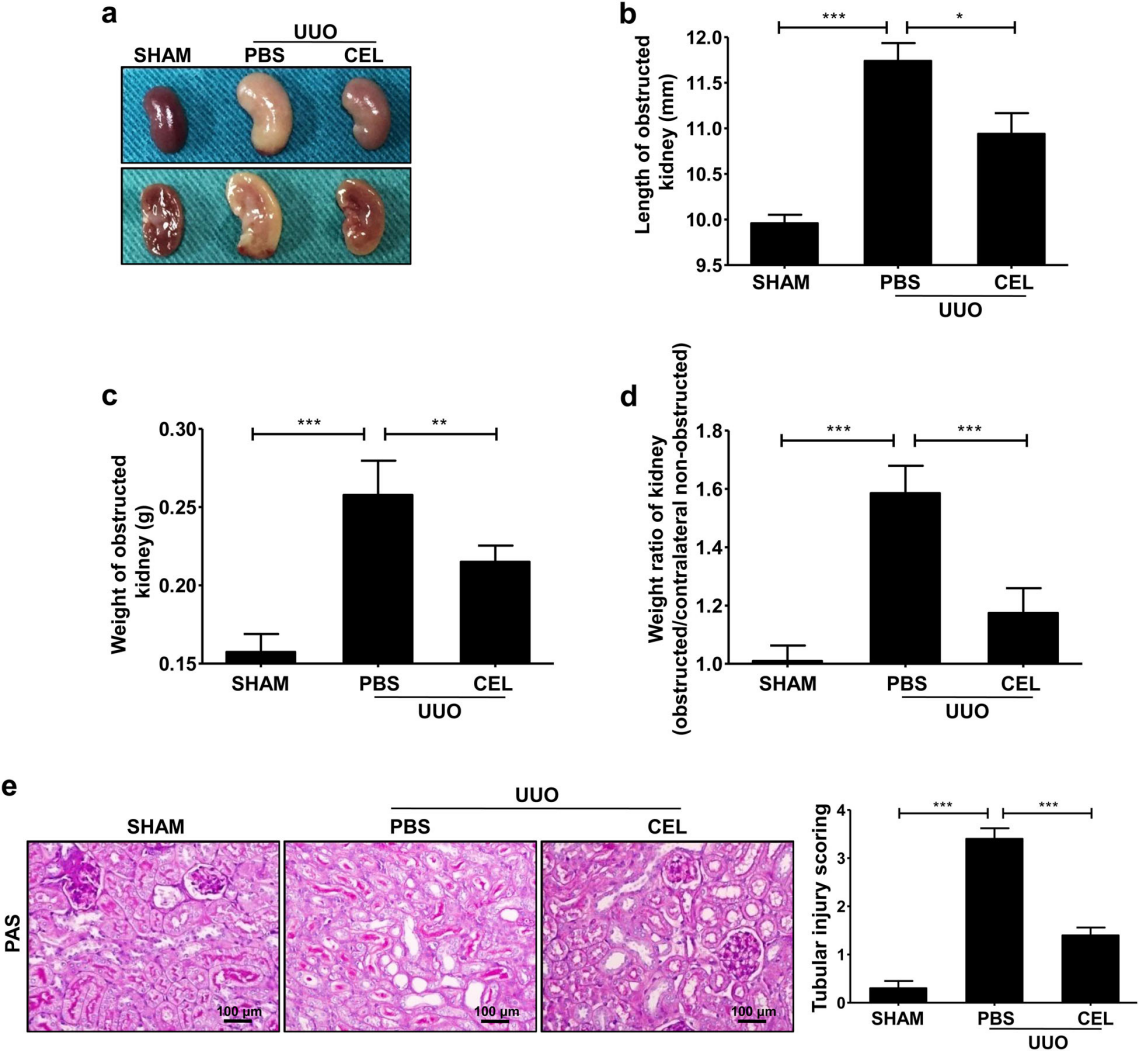
Celastrol improves UUO-induced renal injury. The mice received celastrol (1 mg/kg) daily for 7 days from the day of surgery. The vehicle-treated mice were treated with an equal volume of PBS, and the sham group was used as the control for UUO. Kidney tissues were collected 7 days after UUO. **a** The gross-morphological images of kidney tissue from each group mice. **b** Length and **c** weight of the obstructed kidney, and **d** weight ratio of the kidney (obstructed/contralateral non-obstructed kidney) from each group mice were measured. **e** Representative micrographs show kidney tubular injury determined using PAS staining and quantification of tubular injury scoring of kidneys in different groups of mice. All values are represented as mean ± SEM. Scale bar = 100 μm. *n* = 5/group. *P *<* 0.05, **P *<* 0.01, and ***P *<* 0.001

To explore the effects of celastrol treatment on renal fibrosis, we used a UUO-induced mouse renal fibrosis model. We discovered that the cortex of the obstructed kidney in celastrol-treated mice was thicker and had a richer blood supply than that of the phosphate-buffered saline (PBS)-treated mice 7 days after the UUO surgery (Fig. 1a). Moreover, celastrol administration significantly reversed the UUO-induced increase in the length and weight of the mouse kidneys (Fig. 1b–d). In addition, the periodic acid Schiff (PAS) staining showed that celastrol significantly reduced tubule brush border disruption and tubular atrophy, indicating the attenuation of UUO-induced tubular injury (Fig. 1e).

**Fig. 2:**
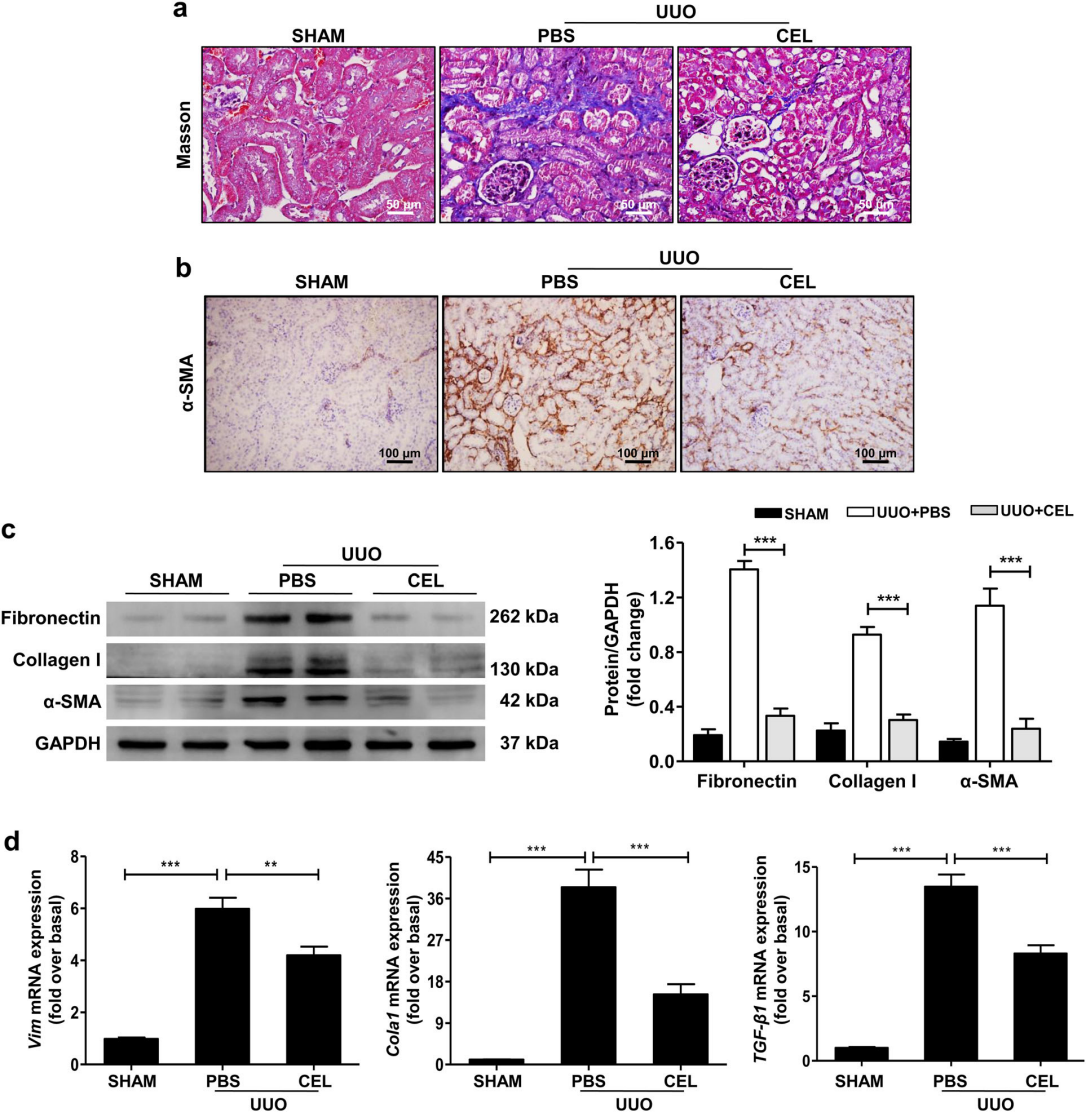
Celastrol suppresses renal fibrosis in UUO mouse model. The mice received celastrol (1 mg/kg) daily for 7 days from the day of surgery. The vehicle-treated mice were treated with an equal volume of PBS, and the sham group was used as the control for UUO. Kidney tissues were collected 7 days after UUO. **a** Masson’s trichrome staining of kidney tissues sections. Scale bar = 50 μm. **b** Representative micrographs and quantitative assessment of the expression and distribution of α-SMA in kidney tissues using immunohistochemical staining. Scale bar = 100 μm. **c** Western blot analyses of renal fibronectin, collagen I, and α-SMA protein in kidney tissues. Representative western blot and quantitative data were presented. **d** Expression of the pro-fibrotic mediators *Vim*, *Cola1*, and *TGF-β1* was measured using qRT-PCR. All values are represented as mean ± SEM. *n* = 5/group. **P *<* 0.01 and ***P *<* 0.001

Furthermore, celastrol treatment led to a remarkable reduction in collagen deposition by Masson’s trichrome staining (Fig. 2a); the expression of fibronectin, collagen I, and α-smooth muscle actin (α-SMA, the marker of myofibroblast) by immunohistochemical staining and western blot assay (Fig. 2b, c); and the mRNA levels of pro-fibrotic markers *Vim*, *Cola1*, and *TGF-β1* by quantitative real-time (qRT-PCR) detecting (Fig. 2d). Celastrol showed similar effects on renal fibrosis in the FA-induced mouse renal fibrosis model (Supplementary Fig. S1a–d). These results indicate that celastrol observably improves renal fibrosis.

### Celastrol treatment reduces UUO-induced inflammatory responses in mice

**Fig. 3:**
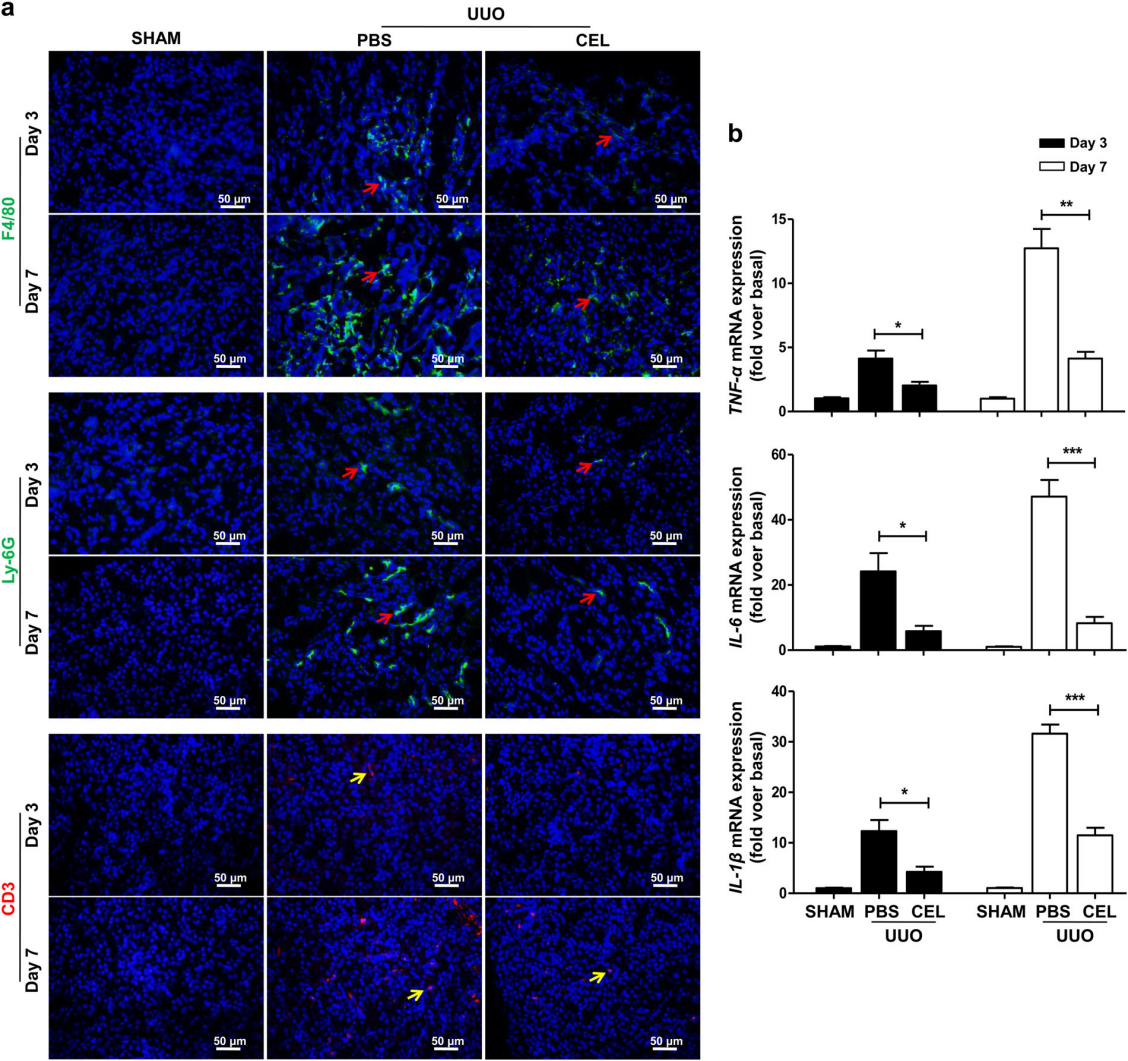
Celastrol inhibits UUO-induced inflammatory responses. The mice received celastrol (1 mg/kg) daily from the day of surgery. The vehicle-treated mice were treated with an equal volume of PBS, and the sham group was the control for UUO. Kidney tissues were collected at days 3 and 7 after UUO. **a** Representative micrographs of the infiltration of macrophages (F4/80^+^, red arrows), neutrophils (Ly-6G^+^, red arrows) and T lymphocytes (CD3^+^, yellow arrows) in kidney tissues using immunohistochemical staining. **b** The expression of pro-inflammatory cytokines *TNF-α*, *IL-6*, and *IL-1β* in kidney tissues was measured using qRT-PCR. All values are represented as mean ± SEM. Scale bar = 50 μm. *n* = 5/group. *P *<* 0.05, **P *<* 0.01, and ***P *<* 0.001

Persistent inflammatory responses play a vital role in renal fibrosis progression^21^. To investigate whether celastrol exerts an anti-inflammatory effect, the inflammatory cell infiltration and pro-inflammatory cytokine expression in the kidney tissues in mice were analyzed using an immunofluorescence assay and qRT-PCR at days 3 and 7 after UUO surgery. The results showed that celastrol treatment remarkably reduced the infiltration of macrophages (F4/80^+^), neutrophils (Ly-6G^+^), and T lymphocytes (CD3^+^) in kidney tissues (Fig. 3a). Furthermore, the levels of the pro-inflammatory cytokines *TNF-α*, *IL-6*, and *IL-1β* mRNA in kidney tissues of celastrol-treated mice were significantly lower than that of the PBS-treated control mice (Fig. 3b). These results demonstrate that the reduced renal fibrosis in celastrol-treated mice may be associated with the reduced inflammatory response.

### Celastrol upregulates CB2R expression during development of renal fibrosis

**Fig. 4:**
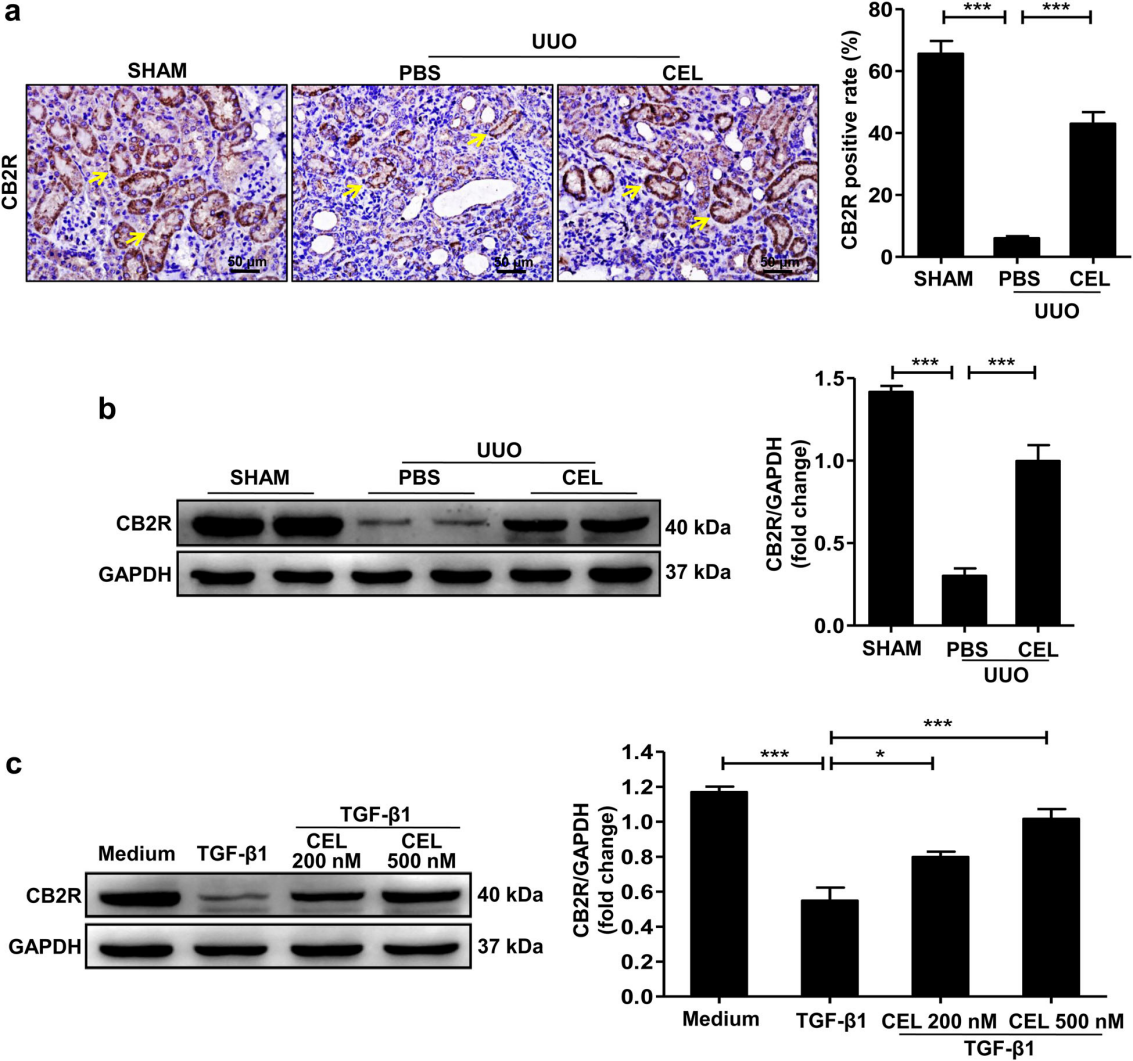
Celastrol upregulates CB2R expression during development of renal fibrosis. **a**, **b** Mice received celastrol (1 mg/kg) daily for 7 days from the day of surgery. The vehicle-treated mice were treated with an equal volume of PBS, and the sham group was the control for UUO. Kidney tissues were collected at 7 days after UUO. Representative results of immunohistochemistry and western blot of CB2R expression in the obstructed kidney tissue. Quantitative data were presented. **c** Serum-starved HK-2 cells were pretreated with or without indicated concentrations of celastrol for 1 h and then stimulated with TGF-β1 (10 ng/ml) for 24 h. Representative western blots and quantification data of CB2R expression in HK-2 cells were presented. The medium group was used as the control for TGF-β1 treatment. Results are representative of three replicate experiments. All values are represented as mean ± SEM. Scale bar = 50 μm. *n* = 5/group. *P *<* 0.05 and ***P *<* 0.001

Activation of CB2R reduces inflammation in various inflammatory-associated diseases including nephropathy and cystitis^15,17^, and blunts the development of UUO-induced renal fibrosis^20,22^. To explore the relationship between celastrol treatment and CB2R function, we measured CB2R expression levels in kidney tissues of mice after UUO surgery. CB2R expression was downregulated in a time-dependent manner in the UUO-induced obstructed mouse kidneys (Supplementary Fig. S2). However, celastrol treatment markedly inhibited the UUO-induced downregulation of CB2R expression, as shown using both the immunohistochemistry and western blot (Fig. 4a, b). Celastrol similarly regulated CB2R expression in the FA-induced renal fibrosis mouse model (Supplementary Fig. S3).

Tubular epithelial cells (TECs) play a key role in renal fibrosis^23,24^, and CB2R primarily localized on the TECs, as shown in Fig. 4a (yellow arrows) and Supplementary Fig. S4. Consistent with the in vivo results, the typical cobblestone-like morphology of TECs HK-2 was changed into a long spindle fibroblast-like morphology after TGF-β1 stimulation for 48 h (Supplementary Fig. S5a, b). Celastrol treatment significantly attenuated the TGF-β1-induced morphological changes of HK-2 cells (Supplementary Fig. S5c, d). Moreover, TGF-β1-induced downregulation of CB2R expression was significantly reversed by celastrol administration in HK-2 cells (Fig. 4c). These results suggest that celastrol upregulates the anti-fibrotic factor CB2R expression during renal fibrosis pathogenesis.

**Fig. 5:**
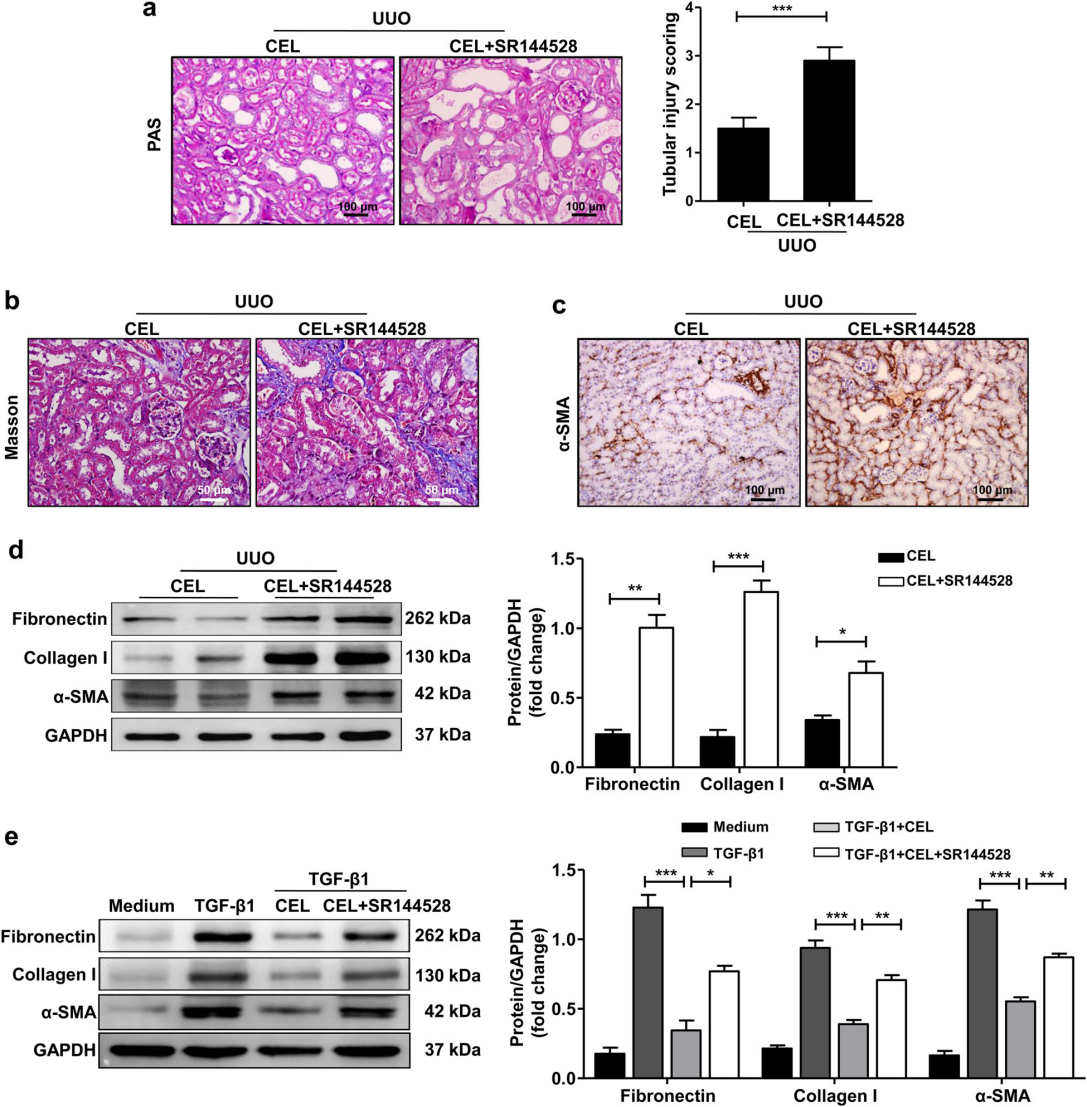
CB2R antagonist SR144528 abolishes anti-renal fibrosis effects of celastrol. **a**–**d** Mice were treated with celastrol (1 mg/kg) by intraperitoneal injection immediately after UUO surgery. The celastrol+SR144528 group was additionally pretreated with SR144528 (1 mg/kg) 1 h before the celastrol injection. Both celastrol and SR144528 were administered daily. Mice were killed 7 days after UUO and kidney tissues were collected. **a** Representative micrographs show kidney injury using PAS staining and quantification of tubular injury scoring in different groups of mice. Bar = 100 μm. **b** Masson’s trichrome staining of kidney tissues sections. Bar = 50 μm. **c** Representative micrographs of the expression and distribution of α-SMA in kidney tissues using immunohistochemical staining. Bar = 100 μm. **d** Western blot analyses of renal fibronectin, collagen I, and α-SMA protein in kidney tissues. Representative western blot and quantitative data were presented. **e** Serum-starved HK-2 cells were pretreated with or without celastrol (500 nM) for 1 h and then stimulated with TGF-β1 (10 ng/ml) for 24 h. The celastrol+SR144528 group was additionally pretreated with SR144528 (1 μM) 30 min before the celastrol administration. Representative western blot analysis and quantification of fibronectin, collagen I, and α-SMA protein expression in HK-2 cells were presented. The medium group was used as the control for TGF-β1 treatment. Results are representative of three replicate experiments. All values are represented as mean ± SEM. *n* = 5 /group. *P *<* 0.05, **P *<* 0.01, and ***P *<* 0.001

To further explore the function of CB2R in the celastrol-mediated anti-fibrotic effect, we used the CB2R antagonist SR144528 to block CB2R endogenous activity. Consistent with a previous study^20^, SR144528 treatment further aggravated UUO-induced renal fibrosis, but celastrol partly reversed this effect (Supplementary Fig. S6a–c). Moreover, SR144528 administration abolished the celastrol-mediated attenuation of UUO-induced tubular injury (Fig. 5a) and the anti-fibrotic effects, as evidenced by the increased collagen deposition and expression of fibronectin, collagen I, and α-SMA in both UUO-induced and FA-induced damaged kidney tissues (Fig. 5b–d, Supplementary Fig. S1b–d). Similarly, the effects of celastrol on the TGF-β1-induced expression of fibronectin, collagen I, and α-SMA in HK-2 cells were remarkably reversed by SR144528 treatment (Fig. 5e). These data indicate that celastrol attenuates the development of renal fibrosis by upregulating the expression of the anti-fibrotic factor CB2R.

### Celastrol upregulates CB2R expression by inhibiting Smad3 signaling activation

**Fig. 6:**
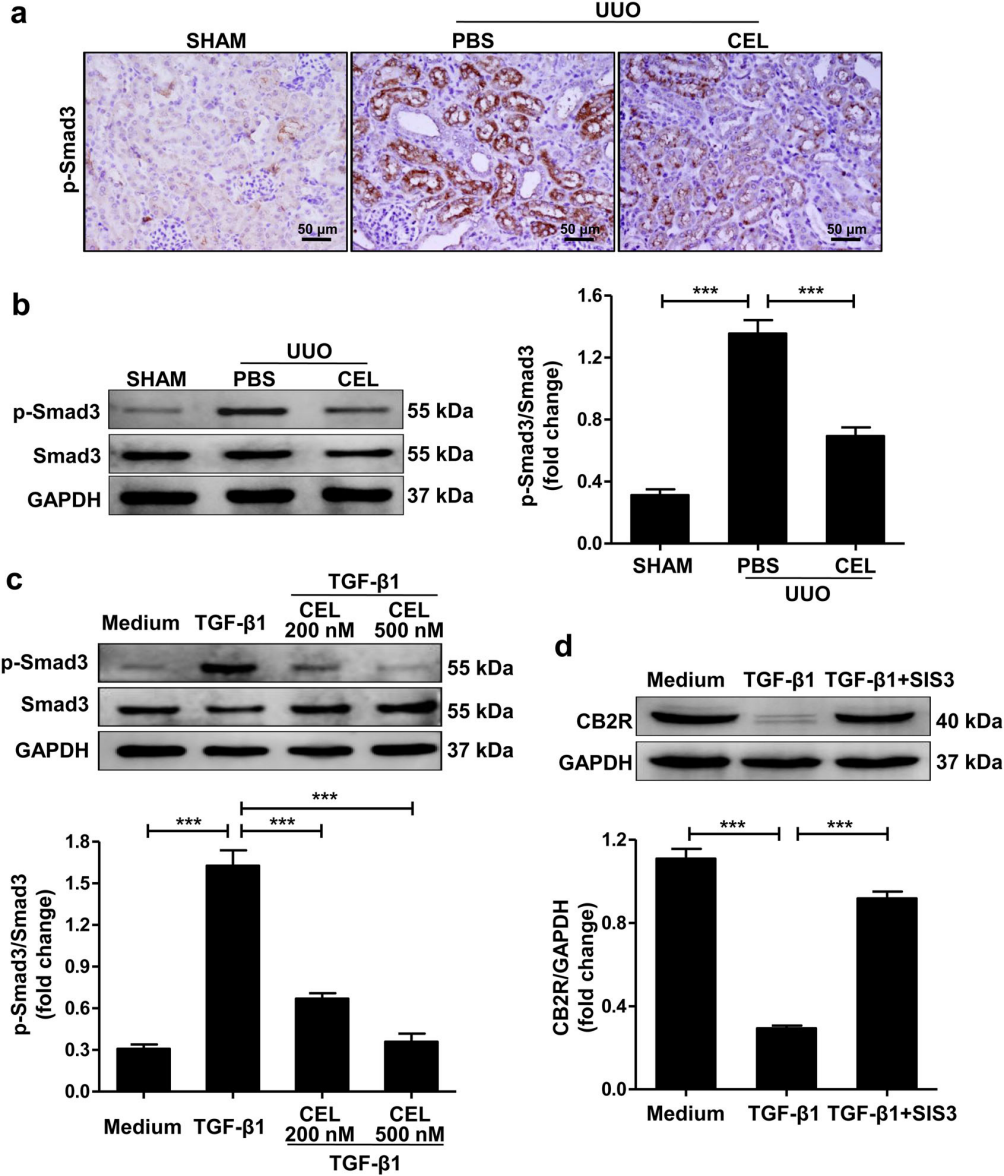
Involvement of Smad3 signaling pathway in celastrol-mediated upregulation of CB2R expression. **a**, **b** Mice received celastrol (1 mg/kg) daily for 7 days from the day of surgery. The vehicle-treated mice were treated with an equal volume of PBS. Sham group was the control for UUO mice. Kidney tissues were collected 7 days after UUO. Representative results of immunohistochemistry and western blot of p-Smad3 expression in the obstructed kidney tissue. Quantitative data were presented. **c** Serum-starved HK-2 cells were pretreated with or without celastrol (500 nM) for 1 h and then stimulated with TGF-β1 (10 ng/ml) for 1 h. Western blot analyses of Smad3 phosphorylation in HK-2 cells and quantitative data were presented. **d** Serum-starved HK-2 cells were pretreated with or without SIS3 (10 ng/ml) for 1 h and then stimulated with TGF-β1 (10 ng/ml) for 24 h. Western blot analyses of CB2R protein in HK-2 cells and quantitative data were presented. The medium group was used as the control for TGF-β1 treatment. Results are representative of three replicate experiments. All values are represented as mean ± SEM. *n* = 5/group. Scale bar = 50 μm. ***P *<* 0.001

Activation of the Smad3 signaling pathway plays a crucial role in the progression of renal fibrosis^25^. In contrast to the increased CB2R expression, the results of immunohistochemical staining (Fig. 6a) and western blot (Fig. 6b) showed that celastrol markedly inhibited UUO-induced Smad3 phosphorylation in the obstructed kidney of mice. Dual-staining of p-Smad3 and CB2R showed that p-Smad3 was primarily expressed in CB2R-positive cells (Supplementary Fig. S7).

Consistently, in vitro, the TGF-β1-induced Smad3 phosphorylation in HK-2 cells was significantly reduced by celastrol treatment (Fig. 6c). Treatment with SIS3, a specific inhibitor of Smad3 phosphorylation, abolished the TGF-β1-induced downregulation in CB2R expression in HK-2 cells (Fig. 6d). These data indicate that celastrol prevents injury-induced degradation of CB2R by inhibiting p-Smad3 during the development of fibrosis.

## Discussion

Renal fibrosis, the main process involved in the progression of CKD to end-stage renal disease, is a serious clinical problem with high morbidity^26^. However, there are no effective drugs to prevent the development of renal fibrosis, and therefore there is an urgent need to develop satisfactory therapeutic drugs for renal fibrosis. Inflammatory responses play a key role in renal fibrosis^27^, and celastrol has been shown to have a therapeutic effect on inflammation-related diseases^28,29^. Therefore, we evaluated the potential therapeutic effects of celastrol on renal fibrosis using the UUO-induced and FA-induced murine renal fibrosis models. The results showed that celastrol treatment significantly attenuated tissue injury and suppressed inflammatory responses in damaged kidneys. In addition, Masson’s trichrome, immunohistochemical staining and western blot analysis revealed that celastrol inhibited the deposition of ECM components, fibronectin, and collagen I and the accumulation of activated myofibroblast. Similar results were found in our in vitro study. To the best of our knowledge, this is the first study to report that celastrol has a potent therapeutic effect on the progression of renal fibrosis.

As a cannabinoid receptor, CB2R is expressed by a variety of cells such as nonparenchymal liver cells^30^, vascular smooth muscle cells^31^, and cardiomyocytes^14^. CB2R disruption or pharmacological inhibition promoted liver, cardiac, and skin fibrosis, whereas CB2R agonists reduced fibrogenesis^30,32,33^. Recent studies have reported that CB2R was also expressed in renal cortex samples, with abundant expression in proximal tubule cells^34^. Similar to the result in patients with advanced diabetic nephropathy^35^, the expression of CB2R in kidney tissues was significantly downregulated in the UUO- and FA-induced mice and treatment with CB2R antagonist SR144528 further aggravated renal fibrosis. Indeed, modulation of CB2R activity ameliorates the renal damage-associated pathologies in UUO mice^20^. Furthermore, we found that celastrol significantly inhibited UUO- or FA-induced downregulation of the anti-fibrotic factor CB2R expression in kidney tissues of mice, and CB2R antagonist SR144528 abolished the celastrol-induced anti-fibrotic effects, indicating that CB2R participates in the anti-fibrosis process of celastrol. Moreover, CB2R agonist JWH133 promoted CB2R expression and alleviated bleomycin-induced pulmonary fibrosis^36^, while treatment with CB2R antagonist or knock-down of CB2R gene contributed to the liver fibrosis by suppressing activated hepatic stellate cells apoptosis^37^, suggesting that the abundance of CB2R is closely related to its anti-fibrotic function. CB2R signaling exerts the role of anti-fibrosis through modulating TGF-β1/Smad pathway activation, fibroblast proliferation, and the function of immune cells^33,36,38^. Therefore, our findings suggest that celastrol administration protects the mice from renal fibrosis by upregulating the endogenous CB2R expression in kidney tissues.

Smad3 signaling is closely associated with TGF-β-induced renal fibrosis^39^, and its activation promotes renal fibrosis by directly triggering collagen production through its binding to specific promoter regions of collagen genes, and inhibition of ECM degradation^13,40,41^. *Smad3*-deficient mice were protected against UUO-induced renal fibrosis^42^, and Smad3 inhibition observably improved renal fibrosis in type 1 diabetic kidney disease^43^. In this study, we found that the Smad3 pathway was involved in celastrol-mediated upregulation of CB2R expression. This conclusion is supported by the following findings: (I) Celastrol upregulated anti-fibrotic factor CB2R expression in the damaged kidney tissues and TGF-β1-stimulated HK-2 cells. (II) Celastrol administration suppressed Smad3 phosphorylation in vivo and in vitro. (III) The expression of p-Smad3 was primarily in CB2R-positive cells. (IV) SIS3, a specific inhibitor of Smad3 activation, reversed downregulation of CB2R expression. These results indicate that celastrol promotes CB2R expression by inhibiting Smad3 pathway activation.

Collectively, our results provide direct evidence for the anti-fibrotic effect of celastrol during renal fibrosis development. These findings indicate that celastrol is a potential therapeutic drug for patients during the development of renal fibrosis. Moreover, this is the first study to show that celastrol upregulates the expression of the anti-fibrotic factor CB2R by inhibiting Smad3 signaling pathway activation.

## Materials and methods

### Animal model

The male BALB/C mice (6–8-week-old) used in this present study were purchased from the Peking University Animal Center (Beijing, China). UUO-induced renal fibrosis was established as described previously^44^. Briefly, the mice were anesthetized with 1% pentobarbital (10 μl/g), the left ureter was exposed by a midline incision, and then it was obstructed by two-point ligations with 6–0 silk sutures. Sham-operated mice had their ureters exposed, manipulated, and they underwent the same procedure but were not ligated. The mice were randomly divided into the following five groups (*n* = 5 each): (I) sham-operated, (II) UUO+vehicle, (III) UUO+celastrol, (IV) UUO+SR144528, and (V) UUO+celastrol+SR144528 groups. The mice were treated with celastrol (1 mg/kg, Medchemexpress, NJ, USA) by intraperitoneal injection immediately after the UUO surgery. The SR144528 and celastrol+SR144528 groups were pretreated with SR144528 (1 mg/kg, Adooq Bioscience, Irvine, CA, USA) 1 h before the surgery. Both celastrol and SR144528 were administered daily for 7 days from the day of surgery. The vehicle-treated UUO mice were treated with an equal volume of PBS. All the mice were killed at indicated time points and the kidneys were harvested.

FA-induced renal fibrosis was established using a previously described standard protocol^4^. Briefly, the mice were intraperitoneally injected with a single dose of FA (250 mg/kg). Four groups of mice (n = 5 each) were used: (I) Control, (II) FA+vehicle, (III) FA+celastrol, and (IV) FA+celastrol+SR144528 groups. Celastrol (1 mg/kg) and SR144528 (1 mg/kg) were administered by intraperitoneal injections 3 days after the FA injection. The celastrol + SR144528 group was additionally pretreated with SR144528 1 h before the celastrol injection. Both celastrol and SR144528 were administered daily for 21 days, and then all mice were killed. The vehicle-treated FA-induced mice were treated with an equal volume of PBS. All animal studies were approved by the Institutional Animal Care and Use Committee of the Third Military Medical University.

### Cells culture and treatment

HK-2 cells were purchased from the American Type Culture Collection (ATCC, Manassas, VA, USA). The HK-2 cells were cultured in Dulbecco’s modified Eagle’s medium (DMEM)/F12 medium supplemented with 10% fetal bovine serum (ScienCell, Carlsbad, CA, USA), 100 U/ml penicillin/streptomycin (Life Technologies, Grand Island, NY, USA). To determine the appropriate concentration of celastrol for the in vitro experiments, serum-starved HK-2 cells were seeded into 96-well plates and incubated with the indicated amount of celastrol for 24 h and then the cytotoxicity of celastrol was assessed using the cell-counting kit-8 (CCK-8) assay (Dojinodo, Shanghai, China) according to the manufacturer’s instructions. No obvious cell death was observed following incubation with celastrol concentrations as high as 500 nM, but the cell viability was <80% at concentrations up to 1000 nM (Supplementary Fig. S8). Therefore, a concentration range of 0 to 500 nM celastrol was used for the subsequent experiments. For the in vitro studies, serum-starved HK-2 cells were preincubated with or without the indicated amount of celastrol, SR144528 (1 μM), or SIS3 (10 ng/ml; Medchemexpress, USA) for 1 h, followed by stimulation with recombinant murine TGF-β1 (10 ng/ml; R&D Systems, Minneapolis, MN, USA) for indicated time points. The morphology of HK-2 cells was observed by phase-contrast microscopy and the cell lysate was harvested for further study. The medium group was used as the control for TGF-β1 treatment.

### Transient transfection and identification of CB2R antibody specificity

Considering the consistent expression of CB2R on macrophages^45^, the specificity of CB2R antibody was identified by detecting the downregulation of CB2R expression in RAW 264.7 cells after treatment with CB2R siRNA. Briefly, cells were plated at 1 × 10^3^ cells/cm^2^ in complete medium without antibiotics. After 70% confluent, cells were transfected with 10 μM siRNA for CB2R (CB2R siRNA, Santa Cruz, CA, USA) or scramble control siRNA (Scr siRNA, Santa Cruz, CA, USA) using lipofectamine 2000 (Invitrogen, CA, USA) according to the manufacturer protocol. After 6 h, the medium was replaced with complete medium and cells were continued cultured for 48 h. The efficacy of the siRNA on the expression of CB2R was assayed by qRT-PCR (Supplementary Fig. S9a). The specificity of CB2R antibody was assayed by western blot (Supplementary Fig. S9b).

### Histology and immunohistochemical staining

Kidney tissues were fixed in 4% formalin, embedded in paraffin, and cut into sections (4-μm-thick), which were stained with PAS and Masson’s trichrome. The tubular injury level of the PAS-stained tissue was scored by grading the tubular dilatation, brush border loss, and epithelial simplification. The following 0 to 4 grading scale was used for the tubular injury scoring: 0, normal; 1, <25% of the cortex is involved; 2, 25–50% of the cortex is involved; 3, 50–75% of the cortex is involved; and 4, > 75% of the cortex is involved^46^. At least 10 randomly selected cortical fields were observed under the microscope and evaluated for each mouse in a blinded manner. Masson’s trichrome staining was used to assess the collagen deposition in the obstructed kidney tissue.

The immunohistochemical staining was performed as previously described^47^. Briefly, sections mounted on slides were blocked with 5% BSA for 1 h and incubated at 4 °C overnight with primary antibodies against α-SMA (1:150), p-Smad3 (1:200), and CB2R (1:20) (All from Abcam, Cambridge, MA, USA). The slides were then stained with a horseradish peroxidase-conjugated secondary antibody (1:800; Beyotime, Shanghai, China). The results were analyzed using a 3,3’-diaminobenzidine (DAB) assay kit (ZSGB-BIO, Beijing, China). The slides were visualized using a microscope (Olympus BX51, Japan).

### Immunofluorescence staining

Kidney cryosections were fixed with 4% paraformaldehyde for 15 min at 25 °C. After blocking with 5% BSA for 1 h, the slides were immunostained with primary antibodies against CB2R, p-Smad3, F4/80, Ly-6G, CD3, and CK-18 (All diluted 1:100, Abcam, Cambridge, MA, USA), followed by staining with a DyLight- or Cy3-conjugated secondary antibody (1:300; Biolegend, San Diego, CA, USA). The nuclei were stained using Hoechst 33258 (Enzo, Lausen, Switzerland) and the slides were subsequently visualized using fluorescent microscopy with the Olympus BX51 (Japan).

### Western blot

Total proteins were isolated from kidney and HK-2 cells using the radioimmunoprecipitation assay buffer, and protein concentrations were quantified using the bicinchoninic acid (BCA) protein assay kit (Beyotime, Shanghai, China). Protein samples (35 μg/lane) were resolved using sodium dodecyl sulfate-polyacrylamide gel electrophoresis and then transferred onto polyvinylidene difluoride membranes (Beyotime, Shanghai, China). The membranes were incubated overnight at 4 °C with α-SMA (1:200), fibronectin (1:000), collagen I (1:500), CB2R (1:500), p-Smad3 (1:500), and Smad3 (1:500) (All from Abcam, Cambridge, MA, USA) followed by incubation with horseradish peroxidase-conjugated goat anti-mouse or goat anti-rabbit IgG secondary antibodies (1:3000, ZSGB-BIO, Beijing, China). The immunoblots were visualized using the enhanced chemiluminescence western blot detection system (Millipore, Billerica, MA, USA) with glyceraldehyde 3-phosphate dehydrogenase (GAPDH, 1:1000, Beyotime, Shanghai, China) as the loading control.

### Quantitative real-time PCR

Total RNA was extracted from the tissues using the TRIzol reagent (Takara, Tokyo, Japan) according to the manufacturer’s protocol. First-strand cDNA was synthesized using an RT system (Takara, Tokyo, Japan) according to the manufacturer’s instruction, and the cDNA was used for qRT-PCR analyses using SYBR Premix Ex Taq (Takara, Tokyo, Japan). The mRNA levels were normalized to that of the housekeeping gene *GAPDH*. The primer sequences used for the amplification are presented in Supplementary Table S1. All samples were measured in triplicates and differences in gene expression were calculated using the 2^−ΔΔct^ method.

### Statistical analysis

All the data are represented as means ± standard error of the mean (SEM). Results from different groups were analyzed by Student’s *t*-test or one-way analysis of variance (ANOVA) followed by Tukey’s post hoc test, and *P* < 0.05 indicated significance.

## Electronic supplementary material

Celastrol alleviates renal fibrosis by upregulating cannabinoid receptor 2 expression
