# Supplementary material for: Celastrol alleviates renal fibrosis by upregulating cannabinoid receptor 2 expression
Source: Cell Death Dis. 2018 May 22;9(6):601. doi: 10.1038/s41419-018-0666-y (PMC5964092; doi:10.1038/s41419-018-0666-y)
Supplement: Supplementary file 1 — Celastrol alleviates renal fibrosis by upregulating cannabinoid receptor 2 expression [file 41419_2018_666_MOESM1_ESM.pdf]

Celastrol alleviates renal fibrosis by upregulating cannabinoid receptor 2 expression

Ming Tang[1,2], Xu Cao[1], Kun Zhang[1], You Li[1], Quan-you Zheng[1], Gui-qing Li[2], Qian-hui He[1], Shu-jing Li[1], Gui-lian Xu[2,*], and Ke-qin Zhang[1,*]

[1]Department of Nephrology, Southwest Hospital, Army Medical University (Third Military Medical University), Chongqing 400038, China.

[2]Department of Immunology, Army Medical University (Third Military Medical University), Chongqing 400038, China.

*Correspondence: Army Medical University (Third Military Medical University), 30 Gaotanyan Main Street, Shapingba District, Chongqing 400038, PR China. E-mail: xuguilian@tmmu.edu.cn (G.-L.Xu); zhkq@tmmu.edu.cn (K.-Q. Zhang)

Running title: Celastrol improves renal fibrosis

Supplementary information:

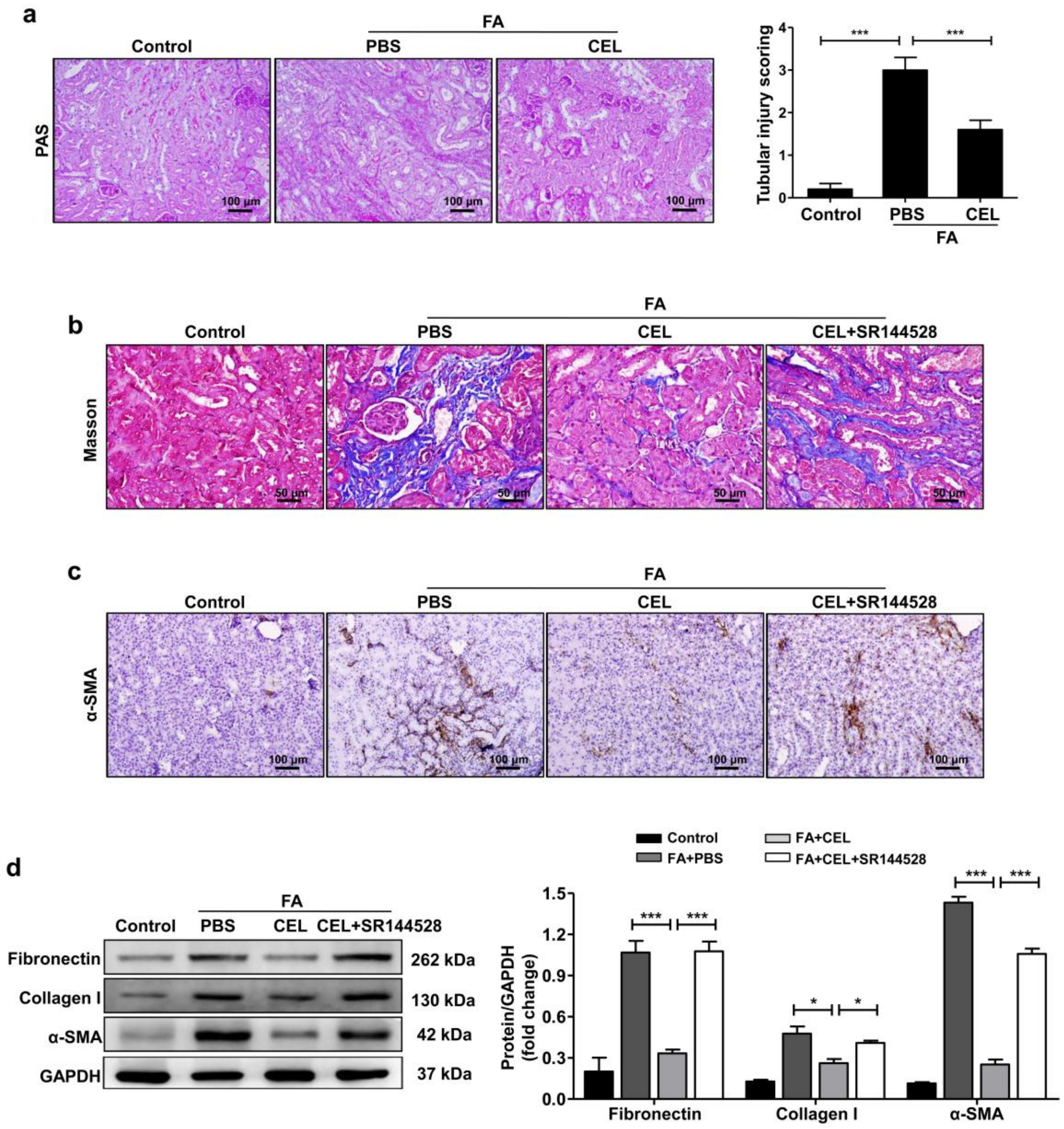

Fig. S1 Celastrol attenuates FA-induced renal fibrosis. Celastrol (1 mg/kg) and SR144528 (1 mg/kg) were administered by intraperitoneal injections 3 days after FA injection. The celastrol + SR144528 group was additionally pretreated with SR144528 1 h before the celastrol injection. Both celastrol and SR144528 were administered daily for 21 days, and then all mice were euthanized. The vehicle-treated mice were treated with an equal volume of PBS. (a) PAS staining was used to show tubular injury, and quantification of tubular injury scoring in different groups of mice

was presented. Scale bar = 100 μm. (b) Masson's trichrome staining of kidney tissues sections. Scale bar = 50 μm. (c) Representative micrographs of the expression of α-SMA in kidney tissues using immunohistochemical staining. Scale bar = 100 μm. (d) Western blot analyses of fibronectin, collagen I, and α-SMA protein in kidney tissues and quantitative data were presented. All values are represented as mean ± SEM. n = 5/group. $^{*}P < 0.05$ and $^{***}P < 0.001$.

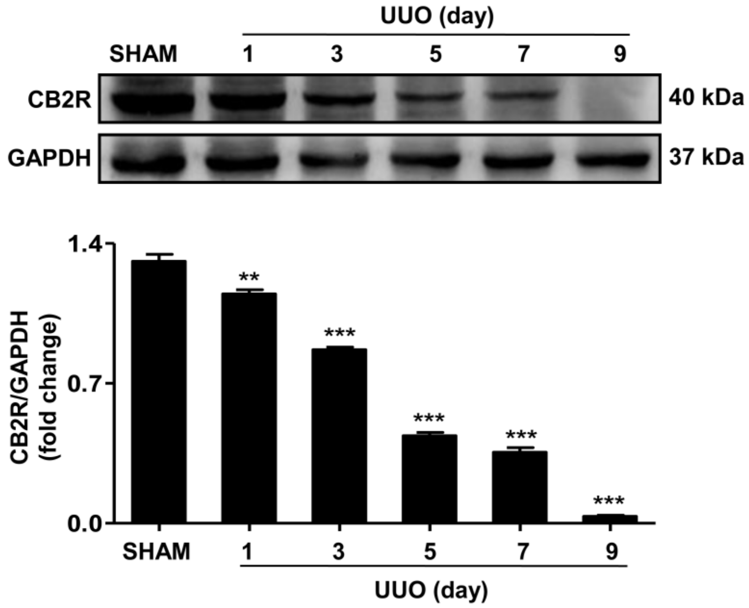

Fig. S2 CB2R expression in UUO-induced obstructed kidney tissues. Mice were euthanized on days 1, 3, 5, 7, and 9 after UUO surgery. Sham group was the control for UUO. Western blot analyses of CB2R expression in the obstructed kidney tissue at the indicated time points and quantitative data were presented. $^{**}P < 0.01$ and $^{***}P < 0.001$ versus sham group.

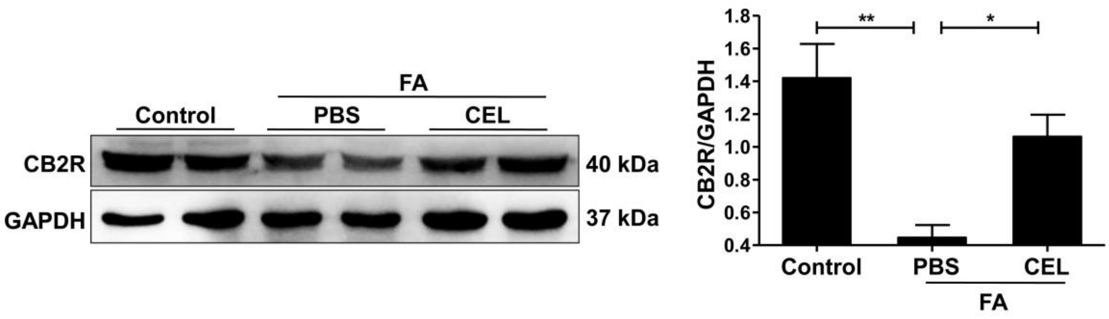

Fig. S3 Effect of celastrol treatment on CB2R expression of FA-induced damaged kidney tissues in mice. Celastrol (1 mg/kg) was administered by intraperitoneal injection 3 days after FA injection. Celastrol was administered daily for 21 days, and then all mice were euthanized. The vehicle-treated mice were treated with an equal volume of PBS. Western blot analyses of CB2R expression in FA-induced damaged kidney tissues, and quantitative data were presented. All values are represented as mean ± SEM. n = 5/group. $^{*}P < 0.05$ and $^{**}P < 0.01$.

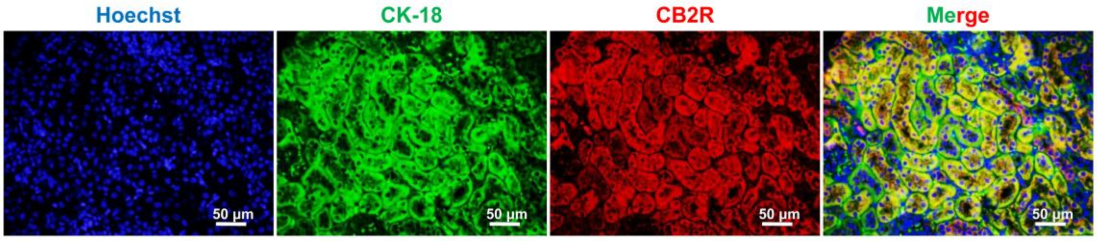

Fig. S4 Expression of CB2R and CK-18 in kidney tissues. Kidney tissues were collected from sham group mice. The location of CB2R (red) and CK-18 (a marker of epithelial cells, green) in kidney tissues was assayed by immunofluorescence. Scale bar = 50 μm.

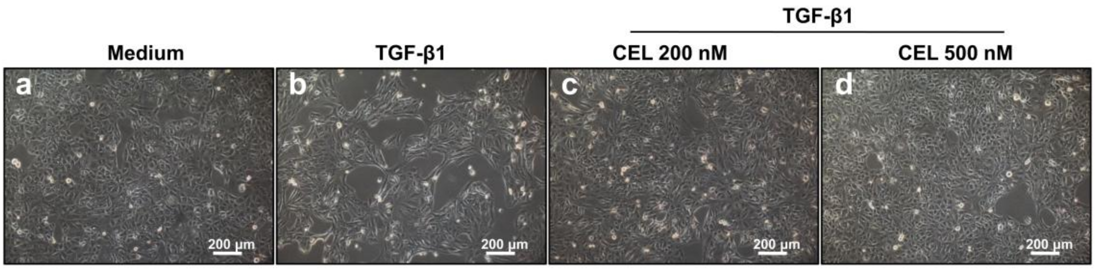

Fig. S5 Effect of celastrol treatment on the morphology of TGF-β1- stimulated HK-2 cells. (a–d) Serum-starved HK-2 cells were preincubated with or without the indicated amount of celastrol for 1 h, followed by stimulation with recombinant murine TGF-β1 (10 ng/ml) for 48 h. The morphological changes of HK-2 cells were observed by phase-contrast microscopy. The medium was used as the negative control of TGF-β1 stimulation group. (a) Cells were polygonal or oval, and displayed the typical cobblestone morphology of epithelial cells in the medium group. (b) After TGF-β1 stimulation, cells lost the cell-to-cell connection, and exhibited a long spindle fibroblast-like morphology. (c, d) Celastrol treatment partly reversed the TGF-β1 stimuation-induced morphological changes of HK2 cells. Results are representative of three replicate experiments. Scale bar = 200 μm.

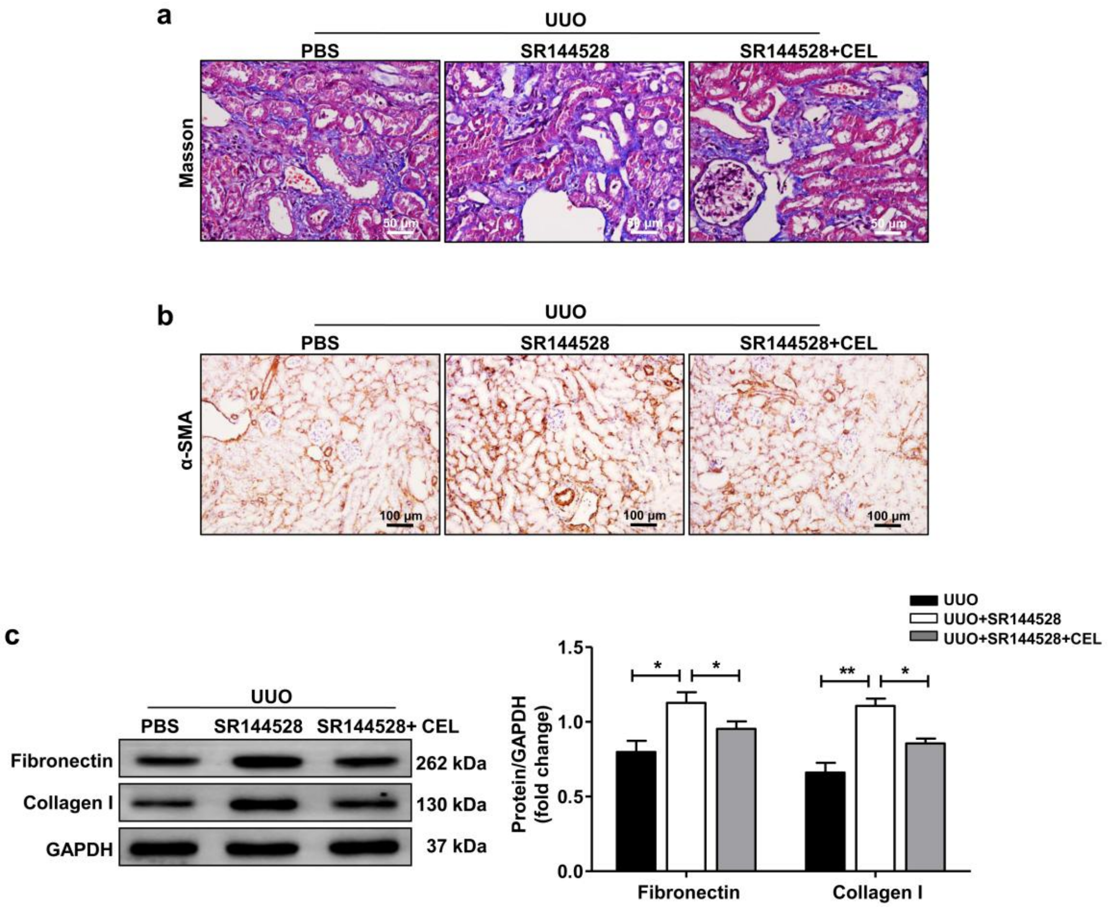

Fig. S6 Effect of SR144528 treatment on UUO-induced renal fibrosis. Mice received SR144528 (1 mg/kg) daily for 7 d after UUO surgery, and PBS treatment was used as the negative control. Kidney tissues were collected at 7 days after UUO. (a) Masson's trichrome staining of kidney tissues sections. Bar = 50 μm. (b) Immunohistochemical staining of α-SMA in kidney tissues. Bar = 100 μm. (c) Western blot analyses of fibronectin and collagen I expression in kidney tissues. Quantitative data were presented at the right. All values are represented as mean ± SEM. n = 5 /group. $^{*}P < 0.05$ and $^{**}P < 0.01$.

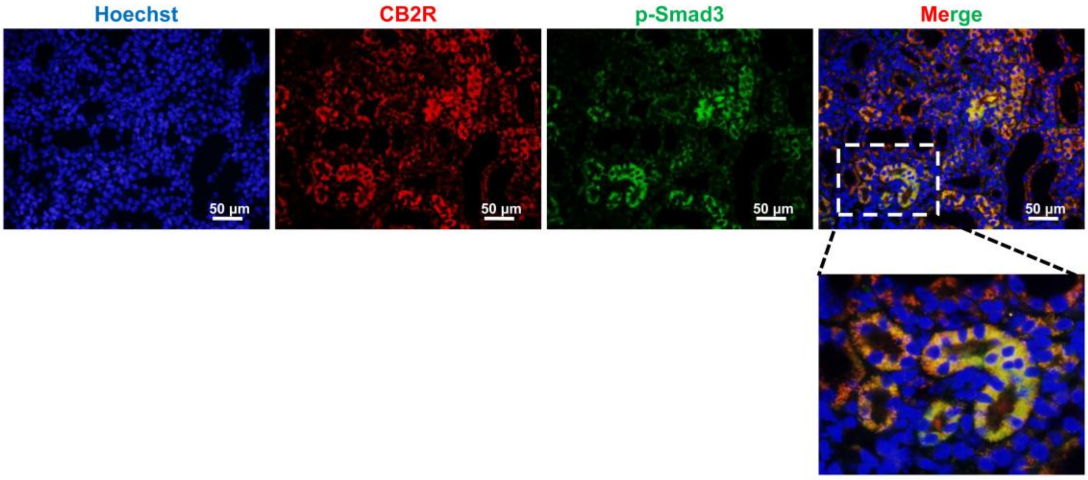

Fig. S7 Expression of CB2R and p-Smad3 in kidney tissues. Kidney tissues were collected after UUO surgery for 7 days in mice. The location of p-Smad3 (green) and CB2R (red) in kidney tissues was assayed by immunofluorescence. Scale bar = 50 μm.

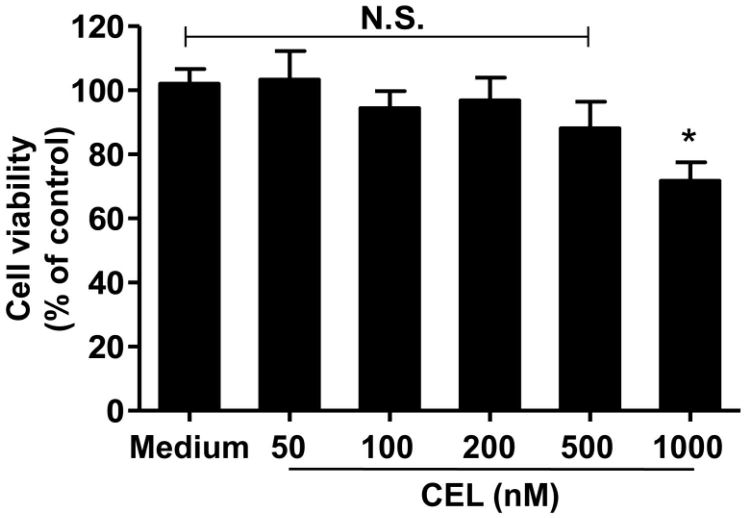

Fig. S8 Effect of different concentrations of celastrol on the viability of HK-2 cells. Serum-starved HK-2 cells were incubated with the indicated amount of celastrol for 24 h, and then cytotoxicity of celastrol was assessed using CCK-8 assay. The medium was used as the negative control of celastrol stimulation. Results are representative of three replicate experiments. All values are represented as mean ±

SEM. N.S.: not significant. $^{*}P < 0.05$ versus medium group.

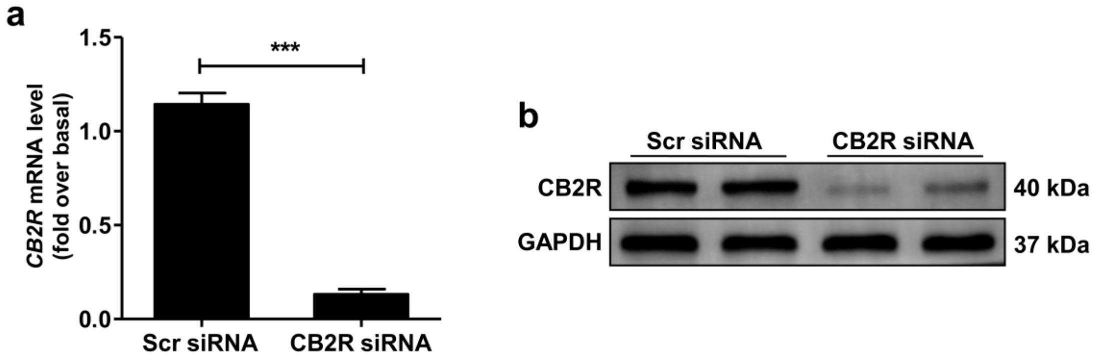

Fig. S9 CB2R expression in RAW264.7 cells after treatment with CB2R siRNA. (a, b) Cells were collected after transfection with CB2R siRNA for 48 h. Scr siRNA treatment was used as the negative control. The expression of CB2R was measured by (a) qRT-PCR and (b) western blot. Results are representative of three replicate experiments. All values are represented as mean ± SEM. $^{***}P < 0.001$.

Supplementary Table S1. Sequences of primers for qRT-PCR.

Gene	Forward primer	Reverse primer
TNF-α	AGGCACTCCCCCAAAAGATG	TTTGCTACGACGTGGGCTAC
IL-1β	TGCCACCTTTTGACAGTGATG	AAGGTCCACGGGAAAGACAC
IL-6	TAGTCCTTCCTACCCCAATTTCC	TTGGTCCTTAGCCACTCCTTC
TGF-β1	GACTCTCCACCTGCAAGACC	GGACTGGCGAGCCTTAGTTT
Cola1	TAAGGGTCCCCAATGGTGAGA	GGGTCCCTCGACTCCTACAT
Vim	TGCCGTTGAAGCTGCTAACTA	CCAGAGGGAGTGAATCCAGATTA
CB2R	TTGCAGGACAGCATACACCC	CAGTGCCACAGTGTTCTTGC

GAPDH	CTCTGCTCCTCCTGTTCGAC	GCGCCCAATACGACCAAATC
